# Water freeze-drying for high-resolution SEM: an accessible strategy for capturing native microorganism morphology without dedicated rapid-freezing systems

**DOI:** 10.3389/fmicb.2026.1870866

**Published:** 2026-08-18

**Authors:** Yoichi Yamada, Wakano Ogawa, Toshinobu Suzaki, Kazuyoshi Murata, Liudmyla Gaponova, Andrii Kolosiuk, Hideki Ishida, Chihong Song

**Affiliations:** 1 School of Pharmacy, Shujitsu University, Okayama, Japan; 2 Department of Biology, Graduate School of Science, Kobe University, Kobe, Japan; 3 National Institute for Physiological Sciences, National Institutes of Natural Sciences, 38 Nishigonaka Myodaiji, Okazaki, Aichi, Japan; 4 Laboratory of Preservation and Renewal of Biodiversity, Institute for Evolutionary Ecology of the National Academy of Sciences of Ukraine, Kyiv, Ukraine; 5 Laboratory of Radiation Technology, Institute of Physics of the National Academy of Sciences of Ukraine, Kyiv, Ukraine; 6 Graduate School of Natural Science and Technology, Shimane University, Nishikawatsu-cho, Matsue, Japan; 7 Department of Convergence Medicine, Pusan National University School of Medicine, Yangsan, Republic of Korea

**Keywords:** *Bacillus subtilis*, cell wall rigidity, dehydration artifacts, *Escherichia coli*, native morphology, scanning electron microscopy (SEM), *Spirostomum ambiguum*, turgor pressure

## Abstract

Conventional scanning electron microscopy (SEM) preparation, relying on chemical dehydration, frequently induces structural artifacts such as shrinkage and distortion. While high-pressure freezing (HPF) offers superior preservation, its accessibility is limited by the requirement for specialized equipment. In this study, we evaluated a water freeze-drying (WFD) method using a general-purpose laboratory freeze-dryer for observing bacteria and protists. Our results demonstrate that WFD effectively preserves morphological integrity by minimizing dehydration-induced stress. Notably, WFD enabled the observation of the highly contractile ciliate *Spirostomum ambiguum* in its natural, extended state without chemical fixation, effectively bypassing the osmotic and physical stresses that typically trigger contraction. Furthermore, WFD revealed significant heterogeneity in *Escherichia coli* polar morphologies; while some cells maintained rounded ends, a substantial subset exhibited remarkably sharp, truncated geometries. In contrast, the Gram-positive bacterium *Bacillus subtilis* consistently retained dome-shaped poles regardless of the drying method, reflecting the superior mechanical rigidity of its cell wall. WFD also revealed substantial heterogeneity in *E. coli* pole morphology, suggesting that conventional dehydration masks physiologically relevant structural diversity. By enabling high-fidelity imaging with standard equipment, the WFD method provides an accessible and robust solution for high-resolution ultrastructural analysis across diverse microbial taxa.

## 1 Introduction

Scanning electron microscopy (SEM) remains an indispensable tool for characterizing the ultrastructure of biological specimens. However, observing biological samples in a vacuum chamber requires rigorous drying to maintain structural integrity. The most conventional preparation protocols involve chemical fixation, followed by dehydration through a graded ethanol series and final drying—typically via critical point drying (CPD) or freeze-drying with organic solvents such as *t*-butyl alcohol (TBA). Unfortunately, these standard dehydration and drying processes frequently induce significant structural artifacts (Hoshina et al., 2020; Ishida et al., 2022a,b, 2023). Organic solvents, in particular, are known to extract membrane lipids and cause substantial shrinkage or cracking of specimen layers (Song and Suzaki, 2013; Song et al., 2017). While high-pressure freezing (HPF) followed by freeze-substitution or cryo-SEM provides excellent structural preservation (Studer et al., 2008), it requires expensive, specialized equipment that is not always accessible to general biological laboratories. Furthermore, certain highly sensitive organisms, such as the contractile ciliate *Spirostomum ambiguum*, can respond to even the subtle mechanical vibrations (Ishida et al., 2026) and pressure changes inherent in the HPF process, leading to undesirable morphological distortions.

In bacteriology, SEM is a powerful method for observing the intricate surface structures of bacteria (Hisada et al., 2023). However, it is crucial to prevent drying-induced deformation, especially when characterizing the bacterial envelope. Many antimicrobial compounds target the cell membrane, and direct observation via electron microscopy is the most critical approach for demonstrating membrane damage (Morita et al., 2015). Conventional preparation methods, however, tend to cause the collapse of bacterial membranes and the surrounding extracellular polysaccharides (EPS) (Nishida et al., 2025). Such artifacts during sample preparation pose a serious challenge, as they may lead to misinterpretations or erroneous speculations regarding the mechanisms of action of antimicrobial agents.

To address these challenges, we have evaluated the water freeze-drying (WFD) method as a solvent-free preparation technique, demonstrating its efficacy for eukaryotic microorganisms and animal tissues (Suzaki et al., 1978, Ishida et al., 2026). While that study introduced the fundamental advantages of bypassing organic solvent dehydration, the present study significantly expands the scope of WFD by establishing a standardized protocol specifically optimized for bacterial cell layers and highly stimulus-sensitive microorganisms.

A primary objective of this work is to demonstrate that WFD effectively preserves the native ultrastructure of *Escherichia coli*—a model bacterium prone to dehydration-induced artifacts—and the highly contractile protozoan *Spirostomum ambiguum*. Furthermore, we show that this high-fidelity preservation is fully compatible with conventional, general-purpose laboratory freeze-dryers, thereby democratizing advanced ultrastructural analysis for a wider range of research environments. By comparing WFD with established cryo-techniques, we define the practical utility and specific strengths of this optimized protocol, providing a robust and accessible solution for broad-scale microbial research.

## 2 Materials and methods

### 2.1 Cells

*Escherichia coli* TG1 was kindly provided by Dr. Tsuchiya (Okayama University). *Bacillus subtilis* NBRC 3134 was obtained from the NITE Biological Resource Center (NBRC), Japan. Cells were cultured in nutrient broth (Nissui Pharmaceutical Co., Ltd., Tokyo, Japan) at 37°C with continuous shaking. *Spirostomum ambiguum* (strain GJ01) was originally collected from a pond in Hiroshima Prefecture, Japan, and maintained as previously described (Ishida et al., 2023).

### 2.2 Specimen preparation for SEM

Bacterial cells (*E. coli* or *B. subtilis*) were initially fixed with 3% glutaraldehyde in 50 mM cacodylate buffer (pH 7.0) containing 20 μM MgCl_2_, and 2 mM sucrose for 30 min at room temperature. The cells were then post-fixed with 0.5% OsO_4_ in the same buffer for 30 min at room temperature. Following fixation, the specimens were dried using either the WFD or TBA method.

**FIGURE 1:**
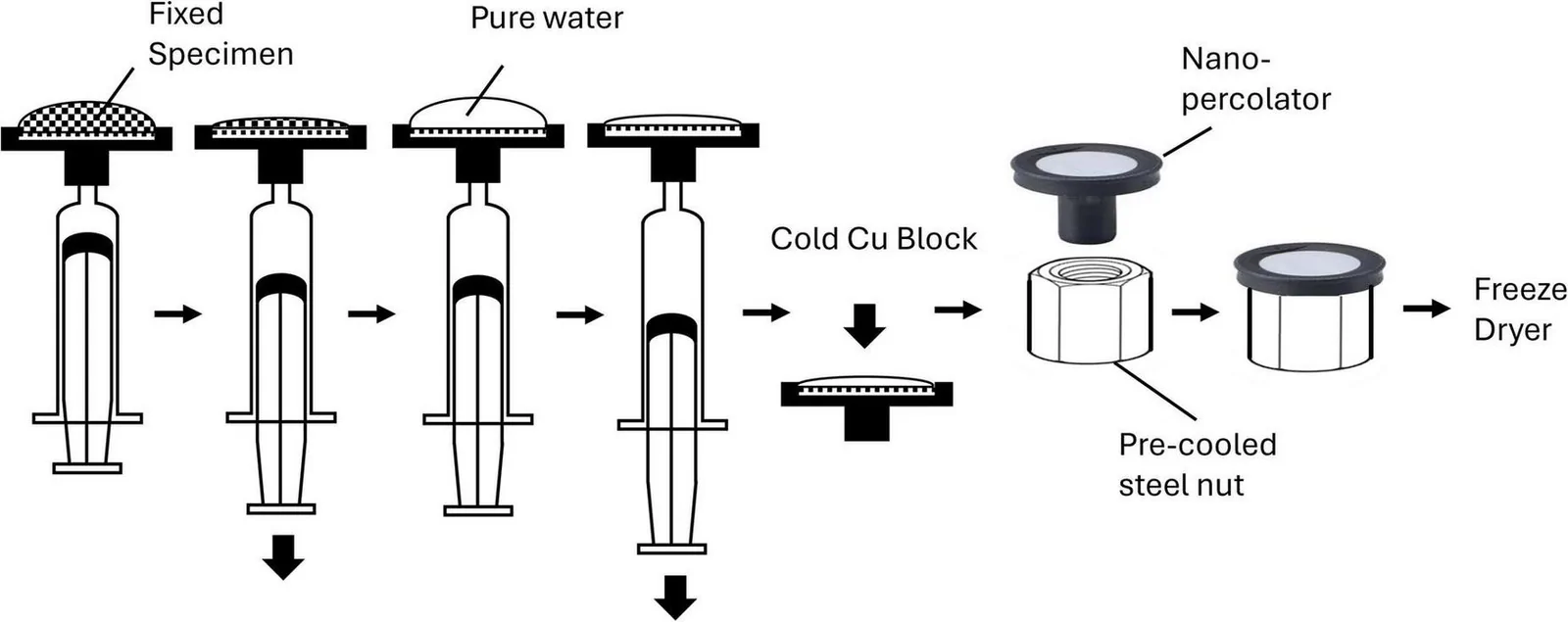
Schematic flowchart of the water freeze-drying (WFD) method. Fixed specimens are placed on a nano-percolator and trapped on its membrane filter. After rinsing with pure water to remove excess salts, the hydrated sample is rapidly frozen by contact with a copper block pre-cooled to –80°C. To maintain the cryo-state during transfer, the nano-percolator is mounted onto a pre-cooled steel nut and subsequently placed into a freeze-dryer. This process can be performed using either a standard laboratory freeze-dryer (e.g., EYELA FDU-2100) or a dedicated t-butyl alcohol freeze-drying apparatus (e.g., VFD-21S).

For the WFD method, the protocol of which is summarized in Figure 1, the fixed cells were concentrated onto a Nano-percolator membrane filter (JEOL, Tokyo, Japan). The concentrated samples were rinsed twice with ultrapure water to remove residual salts. Freezing was performed by slowly lowering a copper block, pre-cooled to –80°C, until it made gentle contact with the stationary specimen surface. Unlike conventional “slam freezing,” which relies on a high-impact collision, this careful and slow movement of the metal block was essential for initiating stable ice formation without inflicting mechanical damage to the specimen. Further details regarding the contact pressure, cooling behavior, and the mechanism of supercooling are described in Ishida et al. (2026). The freezing procedure used for the water freeze-drying method is shown in Supplementary Video S1. The frozen nano-percolator was secured to a pre-cooled (−80°C) steel nut to maintain the temperature. Two freeze-drying apparatuses were used during this study. Quantitative morphometric analysis was performed using specimens dried with a VFD-21S (Vacuum Device Inc., Ibaraki, Japan). In addition, specimens dried with a conventional freeze-dryer (FDU-2100, EYELA, Tokyo, Japan) were examined qualitatively to confirm overall morphological consistency, but these observations were not included in the quantitative analysis. Complete drying was typically achieved within 2–3 h.

For comparison, conventional *t*-butyl alcohol (TBA) freeze-drying (detailed in Ishida et al., 2023) and high-pressure freezing (HPF) (Anderson, 1951) were also employed. In the case of the ciliate *Spirostomum ambiguum*, living cells were frozen directly without prior fixation to evaluate the structural preservation of the WFD method against HPF results.

### 2.3 Morphometric analysis

**FIGURE 2:**
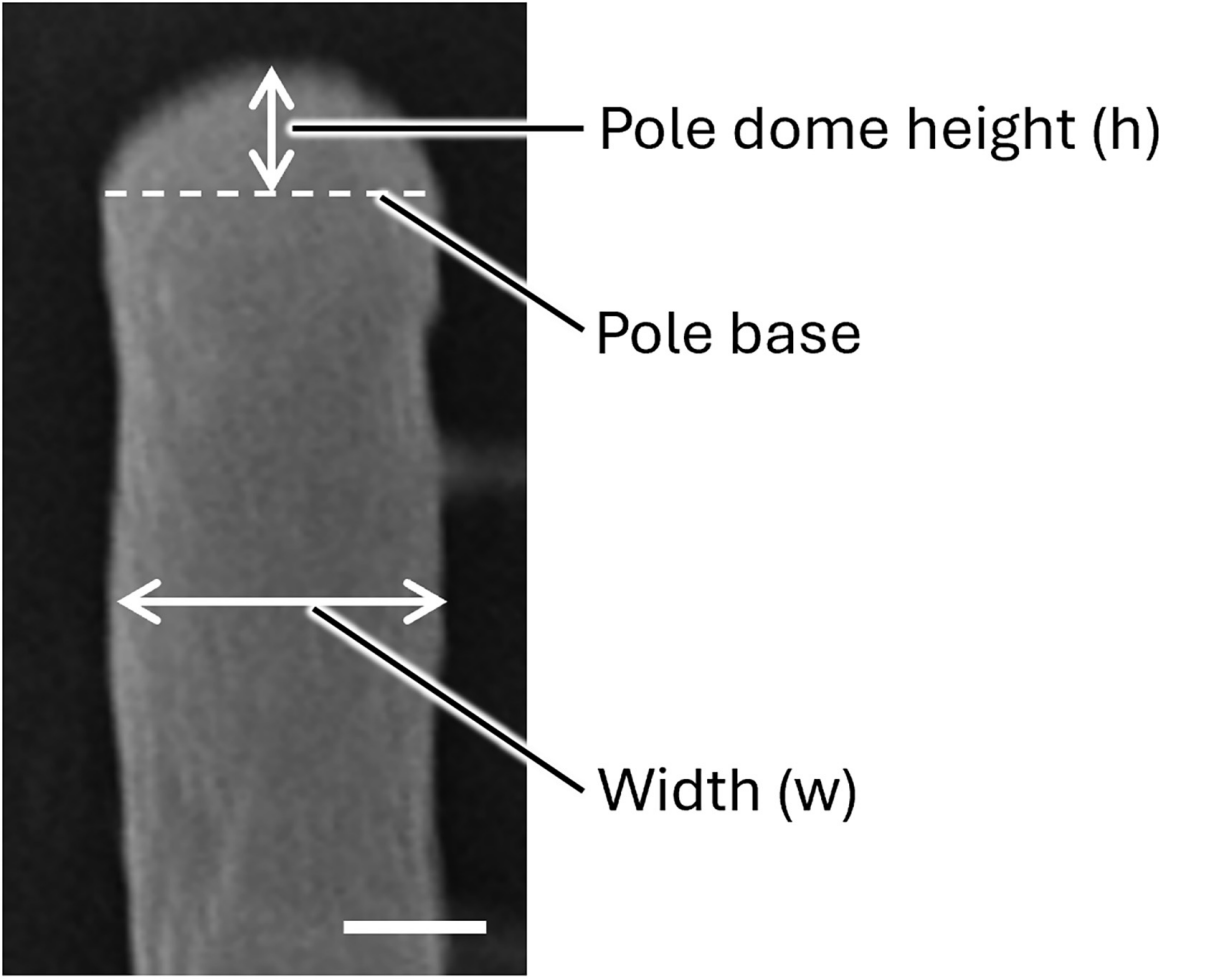
Morphometric parameters used for quantitative analysis of *Escherichia coli* cell morphology. Representative SEM image illustrating the morphometric parameters used in this study. Cell width (w) was measured at the midpoint of the cylindrical region. Pole dome height (h) was defined as the distance between the pole apex and the midpoint of the pole base. The pole base (dashed line) was operationally defined as the position where the cylindrical side wall begins to narrow toward the pole. The pole shape index (PSI) was calculated as 2 h/w, where PSI = 1 corresponds to an ideal hemispherical pole. Scale bar = 0.2 μm.

Morphometric analyses were performed using ImageJ software (National Institutes of Health, Bethesda, MD, United States). The morphometric parameters used in this study are illustrated in Figure 2. To evaluate the effects of specimen preparation on bacterial morphology, cell width, pole dome height, and the pole shape index (PSI) were measured in *E. coli* cells prepared by the WFD and TBA methods. Quantitative measurements were obtained from cells randomly selected from two independently prepared specimen stubs for each preparation method.

Cell width (w) was measured at the midpoint of the cylindrical region of each cell. Pole dome height (h) was defined as the distance between the pole apex and the midpoint of the pole base, where the pole base was operationally defined as the position at which the cylindrical side wall begins to narrow toward the pole (Figure 2).

To quantify pole morphology independently of cell size, the pole shape index (PSI) was calculated as 2 h/w. By definition, PSI = 1 corresponds to an ideal hemispherical pole, whereas values smaller than 1 indicate flatter poles.

Because the *E. coli* TG1 population occasionally contained filamentous cells resulting from abnormal cell division, cell length was excluded from quantitative analysis. Statistical analyses were performed using Welch’s two-sample *t*-test (two-tailed), and differences were considered statistically significant at *p* < 0.05.

## 3 Results

### 3.1 Water freeze-drying preserves the overall morphology of *E. coli*

**FIGURE 3:**
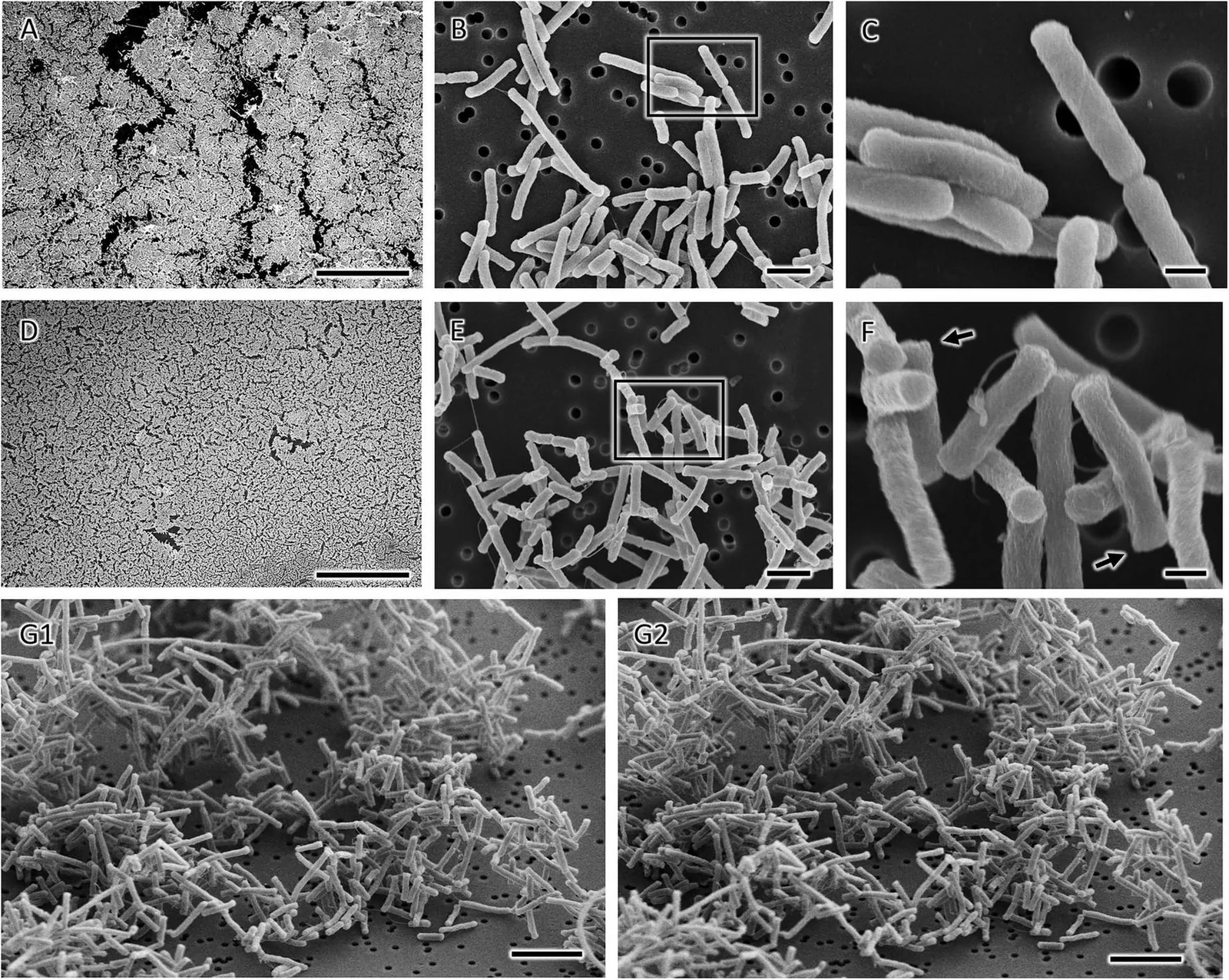
Morphological comparison of *E. coli* prepared by conventional TBA freeze-drying and the WFD method. **(A–C)** Specimens prepared by the conventional *t*-butyl alcohol (TBA) freeze-drying method. At low magnification **(A)**, distinct cracks are visible within the bacterial layer, indicating significant macro-scale specimen shrinkage. High-magnification images **(B,C)** reveal that the cell poles exhibit a characteristic rounded, dome-shaped morphology, which is likely associated with shrinkage during ethanol dehydration and the subsequent redistribution of internal turgor pressure. **(C)** Is an enlarged view of the boxed area in **(B)**. **(D–F)** Specimens prepared by the water freeze-drying (WFD) method (as described in Figure 1) using a standard laboratory freeze-dryer (FDU-2100). In contrast to the TBA method, the low-magnification image **(D)** shows a continuous, intact cell layer without evident cracking or shrinkage. High-magnification observations **(E,F),** reveal heterogeneity in the polar morphologies; while some cells retain rounded poles, others exhibit remarkably sharp, truncated ends (arrows). These distinct shapes may reflect transient physiological states, such as those immediately following cell division (Reshes et al., 2008), which were captured by the rapid immobilization of the WFD process. **(F)** An enlarged view of the boxed area in **(E)**. **(G1,G2)** Stereo-pair micrographs of an *E. coli* specimen prepared by the WFD method. The images show the same field of view with the sample stage tilted at 60° and a 10° difference in rotation angle. Stereo-pairs are arranged for parallel viewing. In all micrographs, the small black circular structures visible in the background represent the pores of the membrane filter. Scale bars: 50 μm for **(A,D)**; 2 μm for **(B,E)**; 500 nm for **(C,F)**; 5 μm for **(G1,G2)**.

We first compared the structural integrity of *E. coli* cell layers prepared by the conventional drying method and the proposed WFD method. In the specimens prepared by the conventional t-butyl alcohol (TBA) freeze-drying method, significant cracking and shrinkage were observed throughout the cell layer on the filter (Figure 3A). This is a typical artifact resulting from the ethanol dehydration and subsequent drying processes. In contrast, the cell layers prepared by the WFD method remained remarkably intact, with no noticeable cracking or large-scale shrinkage (Figure 3D). These results demonstrate that the WFD method, which bypasses organic solvent dehydration, significantly reduces structural artifacts in bacterial specimens.

### 3.2 Morphometric analysis of *E. coli* cell morphology

The WFD method enabled the simultaneous observation of a large number of intact cells, revealing significant morphological heterogeneity at the *E. coli* cell poles that is not evident in conventional preparations. In the specimens prepared by the TBA method, high-magnification images show that nearly all cells exhibit uniform, rounded, dome-shaped poles (Figures 3B,C).

In contrast, the WFD method captured a diverse range of polar geometries within the same population. While some cells in the WFD samples retained the conventional domed appearance (Figure 3E), an enlarged view (Figure 3F) clearly demonstrates that many cells possess remarkably sharp, truncated ends as if they had been cleanly cut (arrows in Figure 3F). These distinct shapes likely reflect transient physiological states, such as those immediately following cell division (Reshes et al., 2008), which are typically masked by the dehydration-induced artifacts of traditional methods.

**FIGURE 4:**
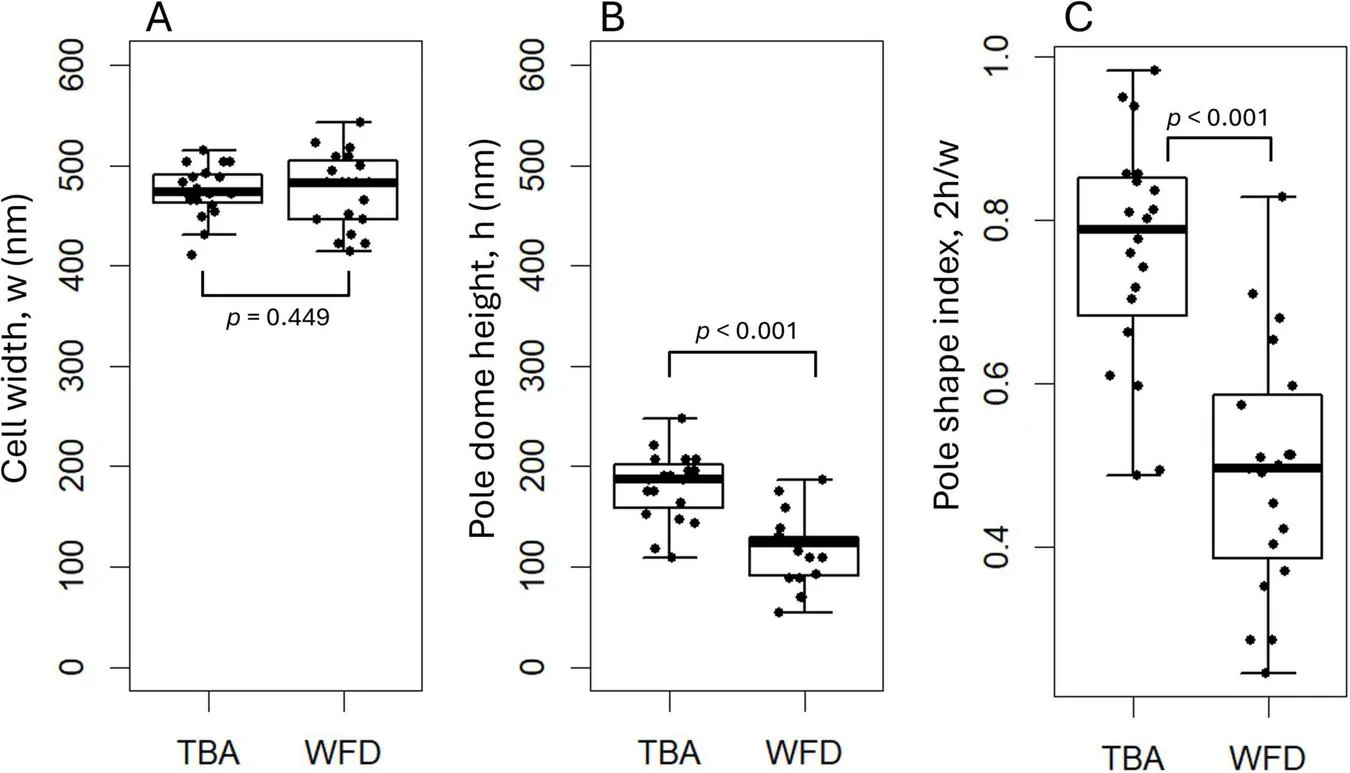
Quantitative comparison of *E. coli* cell morphology following t-butyl alcohol (TBA) freeze-drying and water freeze-drying (WFD). Box-and-whisker plots show the distributions of cell width **(A)**, pole dome height **(B)**, and pole shape index **(C)** (PSI = 2 h/w) measured from individual *E. coli* cells prepared by the TBA and WFD methods. Twenty randomly selected cells from two independently prepared specimen stubs were analyzed for each preparation. Individual measurements are overlaid as black dots. Cell width did not differ significantly between the two preparation methods (Welch’s two-sample *t*-test, *P* = 0.449), whereas both pole dome height and PSI were significantly lower in WFD-prepared cells than in TBA-prepared cells (*P* < 0.001). The lower PSI values indicate that WFD preserves flatter, less dome-shaped cell poles than the conventional TBA method.

Furthermore, the structural integrity of the WFD specimens was confirmed at the population level through stereoscopic analysis. The low-magnification stereo-pair micrographs (Figures 3G1,G2) show a three-dimensional distribution of *E. coli* cells. This observation confirms that the sample did not collapse during the drying process; rather, the cells remained uniformly and cleanly preserved throughout the entire thickness of the original ice layer, from top to bottom. This demonstrates that the WFD protocol provides a more comprehensive and faithful view of the structural diversity within a microbial population compared to the relatively uniform appearance often reported in conventional studies. To quantify these morphological differences, cell width, pole dome height, and the pole shape index (PSI) were measured for individual cells (Figure 4). No significant difference was detected in cell width between the TBA and WFD preparations (*P* = 0.449). In contrast, both pole dome height and PSI were significantly lower in WFD-prepared cells than in TBA-prepared cells (*P* < 0.001 for both), quantitatively confirming that the WFD method preserves flatter cell poles while maintaining overall cell width.

### 3.3 Application of WFD to Gram-positive bacteria

**FIGURE 5:**
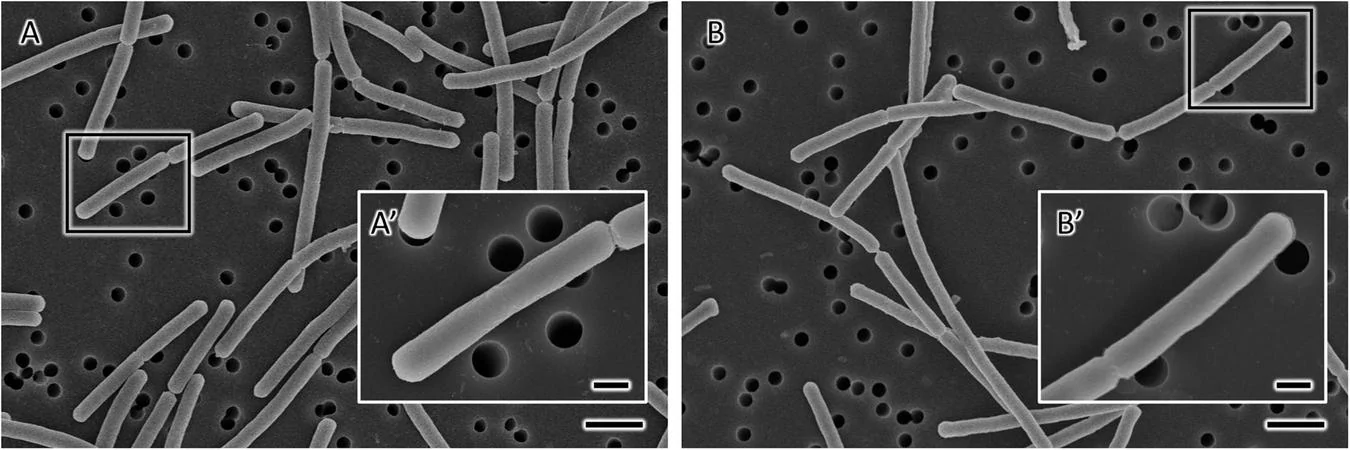
Morphological comparison of the Gram-positive bacterium *Bacillus subtilis* prepared by different freeze-drying methods. SEM images of *B. subtilis* prepared using the conventional **(A)** *t*-butyl alcohol (TBA) freeze-drying method and **(B)** the water freeze-drying (WFD) method. Insets **(A’,B’)** provide magnified views of the boxed areas in **(A,B)**, respectively. In contrast to the results observed for *E. coli*, *B. subtilis* maintains rounded, dome-shaped poles regardless of the preparation technique, reflecting the structural rigidity of its thick Gram-positive cell wall. In all micrographs, the background circular structures represent the pores of the membrane filter. Scale bars: 2 μm for **(A,B)**; 500 nm for the insets **(A’,B’)**.

To examine the effect of the WFD method on Gram-positive bacteria, *B. subtilis* was prepared using both the conventional TBA and WFD methods. As shown in Figure 5, the observations obtained from both techniques were nearly indistinguishable. High-magnification images (Figures 5A’,B’) revealed that the cell poles remained consistently dome-shaped in both the TBA- and WFD-prepared samples, with no significant differences in surface morphology or overall cell dimensions between the two methods.

### 3.4 Application of WFD to unfixed ciliates

**FIGURE 6:**
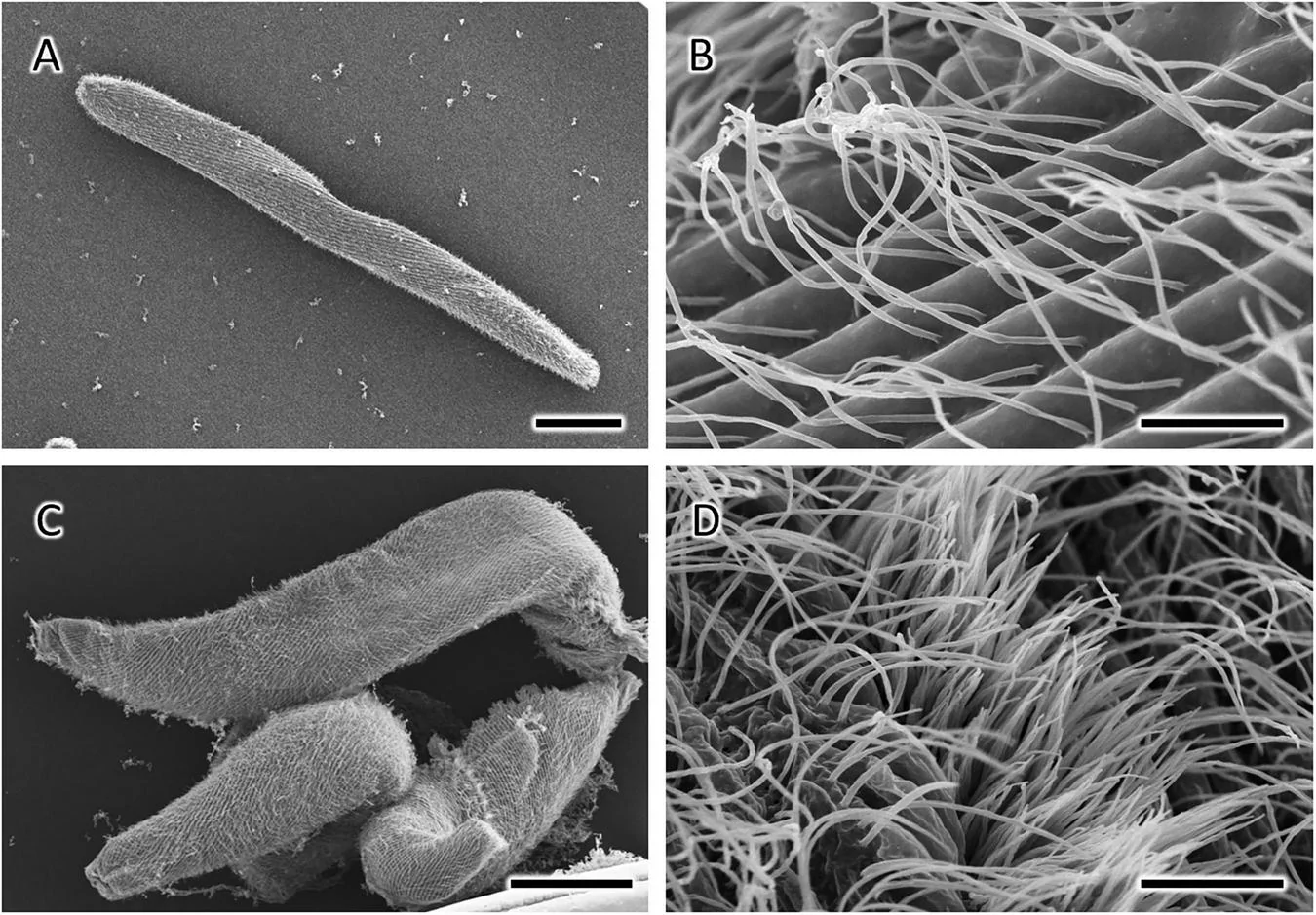
SEM images of unfixed *Spirostomum ambiguum* prepared by two different freezing methods. **(A,B)** Cells prepared by the water freeze-drying method. **(B)** A higher magnification of the cell surface in **(A)**, showing preserved cilia. **(C,D)** Cells prepared by high-pressure freezing (HPF). **(D)** A higher magnification of the cell surface in **(C)**. Scale bars: **(A,C)** 100 μm; **(B,D)** 5 μm.

To evaluate the effect of the freezing process on the contractile state of *Spirostomum ambiguum*, we compared the water freeze-drying method with high-pressure freezing (HPF). When using the water freeze-drying method, the cells were successfully preserved in their extended state without chemical fixation (Figure 6A). This suggests that the method can freeze the cells without triggering their characteristic contraction, which is typically induced by even slight mechanical stimuli. In contrast, cells prepared by HPF exhibited a highly contracted morphology (Figure 6C). This contraction likely occurred during the pressurization process, potentially due to the significant vibrations generated within the HPF apparatus. Despite these differences in the overall cell body shape, high-magnification images revealed that the fine structures of the cilia on the cell surface were well-preserved by both freezing methods (Figures 6B,D).

## 4 Discussion

### 4.1 Characteristics and practical utility of the WFD method

The present study demonstrates that the water freeze-drying (WFD) method is a highly effective and versatile technique for preserving the ultrastructure of biological specimens for SEM. A major advantage of this method is the complete omission of organic solvent dehydration (e.g., ethanol or t-butyl alcohol), which is a notorious source of structural artifacts such as shrinkage and cracking in conventional protocols (Figure 3). These artifacts have long been recognized as a major limitation of SEM specimen preparation and have motivated the development of numerous drying and cryofixation techniques (Tables 1, 2).

**TABLE 1:** Comparison of major cryofixation methods for biological SEM.

Feature/method	HPF	Ambient-pressure RF	WFD
Cooling principle	Rapid cooling under 200 MPa (2,000 bar) to prevent ice growth.	Ultra-fast heat transfer via impact with a cryogenically cooled medium (liquid or solid).	Induction of supercooling via contact with a –80°C copper block.
Vitrification depth	Upto 200 μm.	Very shallow (10–20 μm).	Deep: Up to ∼0.5 mm (500 μm) without ice damage.
Mechanical stimulus	High: Sudden hydrostatic pressure and compression.	High: Physical impact and potential “squashing.”	Negligible: Allows for gentle freezing of sensitive tissues.
Structural preservation	Optimal for organelle-level ultrastructure.	Surface only. Superior for membrane dynamics.	Slightly lower resolution compared to others
Best application	3D reconstruction of resilient tissues.	Ultra-fast events at the specimen surface.	Sensitive or large specimens (e.g., intact microorganisms or fragile organisms).
Time resolution	∼100 ms (Deadtime).	High ( < 10 ms delay).	Moderate (Defined by the onset of supercooling).
Accessibility	Limited (Core facilities only)	Specialized (Lab-built or commercial)	Universal (DIY/Benchtop)
Estimated system cost	Over $250,000	$10,000–$50,000	∼US$7,000 (standard laboratory freeze dryer)
Runningcost	High (liquid N2, specialized consumables)	Moderate (liquid N2/He)	Very low (dry ice, standard vacuum)

HPF, high-pressure freezing; Ambient-pressure RF, ambient-pressure rapid freezing; WFD, water freeze-drying. Ambient-pressure rapid freezing refers to rapid cooling methods performed without high- pressure equipment and does not necessarily involve supercooling induced by controlled copper-block contact.

**TABLE 2:** Comparison of drying methods for biological SEM.

Feature	CPD	TBA-FD	HMDS method	WFD
Drying principle	Phase boundary bypass (Anderson, 1951)	Sublimation (Inoue and Osatake, 1988)	Chemical evaporation (Nation, 1983; Nishida et al., 2025)	Water-based sublimation (Suzaki et al., 1978; Ishida et al., 2022a,b)
Organic solvent dehydration	Required (main cause of shrinkage/lipid extraction)	Required (same as left)	Required (same as left)	Not required (water-based process)
Surface tension	None (phase boundary bypassed at critical point)	None (bypassed via sublimation)	Residual (occurs during liquid- to-gas evaporation)	None (bypassed via sublimation)
Degree of shrinkage	Moderate (Bray et al., 1993)	Moderate (this study)	Moderate to high (Bray et al., 1993)	Minimal (Ishida et al., 2023; this study)
Compatibility with unfixed specimens	Not applicable	Not recommended/practically difficult	Not applicable	Applicable
Equipment	Specialized CPD apparatus (high-pressure vessel & CO_2_)	Freeze dryer	None (fume hood only)	Freeze dryer
	High-pressure physicaL hazards	Relatively low	High (inhalation/neurotoxicity)	Negligible (safe)

CPD, critical point drying; TBA-FD, t-butyl alcohol freeze-drying; HMDS, hexamethyldisilazane; WFD, water freeze-drying.

Our results indicate that the WFD method is not only superior in terms of structural preservation but also remarkably accessible. Historically, high-quality freeze-drying has been perceived to require specialized, expensive EM apparatuses to maintain sublimative conditions. However, the fact that a standard laboratory freeze-dryer yielded results comparable to dedicated systems suggests that this method can be easily adopted by various biological laboratories. This democratization of high-quality sample preparation allows for rapid, high-throughput screening without the significant financial or technical barriers typically associated with advanced cryo-techniques. Furthermore, the elimination of toxic chemicals and the need for complex waste management align with modern laboratory safety and environmental standards (Tables 1, 2).

An additional advantage revealed by the present study is that WFD preserved a broader range of bacterial morphologies than the conventional preparation method. In *Escherichia coli*, WFD not only prevented large-scale shrinkage and cracking but also revealed substantial heterogeneity in cell pole morphology that was largely obscured after conventional TBA preparation. The quantitative significance of these differences is supported by the morphometric analysis presented in Figure 4 and Table 3 and is discussed in the following section.

**TABLE 3:** Quantitative comparison of cell morphology in *Escherichia coli* prepared by t-butyl alcohol (TBA) freeze-drying and water freeze-drying (WFD).

Parameter	TBA freeze-drying	Water freeze-drying	*P-*value
Cell width, *w* (nm)	474 ± 25	476 ± 37	0.449
Pole dome height, *h* (nm)	181 ± 34	117 ± 34	2.9 × 10^–7^
Pole shape index (PSI = 2 *h/w*)	0.762 ± 0.139	0.493 ± 0.151	4.6 × 10^–7^

Values are presented as mean ± SD (*n* = 20 cells). Cell width (*w*), pole dome height (*h*), and the pole shape index (PSI = 2*h*/ *w*) were measured as defined in Figure 2 and the Methods section. Statistical comparisons between the two preparation methods were performed using Welch’s two-sample *t*-test.

### 4.2 Comparative evaluation of cryofixation techniques

Table 1 summarizes a comparison of various cryofixation methods. While high-pressure freezing (HPF) is considered the gold standard for vitrifying thick biological samples (Kelley et al., 2022), it requires substantial capital investment and rigorous, time-sensitive preparation. Furthermore, the rapid pressurization (up to 200 MPa) inherent in HPF can induce significant mechanical artifacts. For instance, McDonald et al. (2007) noted that such sudden pressure changes can trigger instantaneous defensive contractions in highly sensitive organisms. In contrast, our WFD protocol offers a unique balance of high structural fidelity and practical efficiency by operating at ambient pressure. Like conventional ambient-pressure rapid freezing methods, such as plunge freezing and metal-contact freezing, the present protocol does not require high-pressure equipment. However, the freezing mechanism differs in that rapid freezing is initiated by controlled supercooling through the gentle contact of a pre-cooled copper block with the specimen surface, rather than by direct immersion into a cryogen or high-impact metal contact. This controlled initiation of ice nucleation has been characterized previously (Ishida et al., 2026), and the freezing procedure is illustrated in Supplementary Video S1. The successful application of WFD to the highly contractile ciliate *S. ambiguum* (Figure 6) highlights this specific strength. By bypassing the chemical fixation, dehydration, and mechanical compression that trigger osmotic and physical stress, we were able to observe these sensitive cells in their natural, extended state. To ensure the success of this method, preventing ice recrystallization during specimen transfer is critical. The use of a pre-cooled steel nut (Figure 1) as a thermal reservoir proved to be a simple yet effective solution, allowing any laboratory with standard equipment to achieve results that rival more complex and costly cryo-techniques.

### 4.3 Assessment of dehydration and drying protocols

To evaluate the structural integrity achieved in this study, the physical and chemical impacts of various drying protocols are compared in Table 2. Conventional methods, such as CPD (Anderson, 1951), the TBA method (Inoue and Osatake, 1988), and the HMDS method (Nation, 1983; Nishida et al., 2025), inevitably require a graded ethanol dehydration series. As noted by Bray et al. (1993), this extraction of lipids and soluble cellular components is the primary cause of specimen shrinkage.

While the HMDS method is valued for its convenience, it often retains residual surface tension during the final evaporation phase. In delicate structures like cilia, these capillary forces can lead to significant clumping or matting, obscuring fine surface details (Bray et al., 1993). The WFD method, much like CPD, effectively eliminates these forces through sublimation. However, unlike CPD, which operates under high pressure and temperature (the critical point), WFD maintains the sample in a solid, frozen state throughout the drying process, thereby providing a more robust defense against the collapse of sensitive morphologies. In summary, by combining the safety and simplicity of ambient-pressure protocols with the structural fidelity of cryo-sublimation, the WFD method offers a superior and more reliable approach for observing the native morphology of delicate biological specimens.

### 4.4 Morphological heterogeneity of *E. coli* poles

The morphological analysis of *E. coli* provides a noteworthy perspective on the structural preservation of the WFD method (Figure 3). Specimens prepared by the TBA method consistently exhibited dome-shaped, rounded ends, a feature commonly depicted in standard microbiological texts (Hayat, 2000; Watson et al., 1980). In contrast, the WFD method revealed a more heterogeneous population; while many cells maintained rounded poles, a subset of cells displayed remarkably sharp, truncated ends. This qualitative observation is consistent with the morphometric analysis (Table 3), which demonstrated that WFD specimens exhibited a significantly lower cell height and height-to-width ratio than TBA-prepared specimens, despite having comparable cell widths. These findings indicate that the altered pole morphology observed in WFD specimens is accompanied by measurable differences in the three-dimensional geometry of the cells. Qualitatively similar pole morphologies were also observed in WFD specimens prepared using an FDU-2100 freeze dryer (see Materials and methods). The uniform “doming” effect observed in the TBA method could be interpreted as a consequence of cellular shrinkage induced by ethanol dehydration. It is possible that the contraction of the cell surface redistributes internal turgor pressure toward the poles, potentially causing them to bulge. Consequently, the cylindrical morphology observed at the poles of some cells captured by the WFD method—which are not subjected to such dehydration stress—might reflect a transient state of the native *E. coli* structure.

Although Graham et al. (1991) demonstrated that freeze-substitution preserves the near-native state of bacteria, their results primarily depict rounded poles. This discrepancy could stem from the inherent limitations of TEM observations using ultra-thin sections. Given that thin-sectioning captures only a minute fraction of each cell, a sampling bias toward “typical” rounded profiles cannot be ruled out. In contrast, the whole-mount perspective of the WFD method enables the identification of less frequent geometries. We tentatively propose that the coexistence of truncated and domed ends might reflect distinct physiological states, similar to the cell shape dynamics discussed by Reshes et al. (2008). The “truncated” form could potentially represent a transient mechanical state of the cell wall immediately following division, which might be preserved under the rapid stabilization of the WFD process.

However, the observation of cells with *both* ends appearing truncated introduces further complexity. While this phenotype could represent cells captured during a rapid growth phase where both poles are relatively “young” (Reshes et al., 2008), it is also important to consider that these sharp geometries might arise from as-yet-unidentified artifacts specific to the WFD process. Although the WFD method appears to maintain the mechanical rigidity of the peptidoglycan layer effectively, the biological significance of these flat poles remains a subject for further investigation. Because these geometric differences are supported by quantitative morphometric measurements rather than qualitative observations alone, the WFD-specific morphology is unlikely to reflect merely subjective visual impressions. Rather than asserting a definitive native structure, these findings highlight the possibility that the WFD method captures dynamic geometric states previously masked by conventional techniques, although this possibility warrants further investigation.

### 4.5 Species-specific responses to WFD preparation

The observed flattening of *E. coli* cell poles in WFD samples, as opposed to the dome-shaped poles seen in conventional SEM, suggests that the latter may be an artifact of shrinkage-induced turgor pressure. In contrast, the persistent dome-shaped poles in *B. subtilis* under WFD conditions can be attributed to the inherent mechanical rigidity of Gram-positive cell walls. While the peptidoglycan layer of *E. coli* is thin (∼2–6 nm) and susceptible to morphological distortion (Yao et al., 1999), *B. subtilis* possesses a robust, thickened peptidoglycan scaffold (20–40 nm) designed to withstand significantly higher internal turgor pressures (10–20 atm) (Misra et al., 2013; Whatmore and Reed, 1990). During cell division in Gram-positive rods, the hemispherical pole is established as the septum bulges under turgor pressure, eventually becoming a permanent, rigid feature through extensive cross-linking (Li et al., 2021). Consequently, the superior structural integrity of the *B. subtilis* envelope likely preserves its native geometry even during the minor stresses of WFD processing, whereas the more flexible *E. coli* envelope reveals its true, flatter polar morphology when shrinkage is minimized.

### 4.6 Possible applications of WFD in bacterial research

Bacteria have adapted to thrive in nearly every environment on Earth (Whitman et al., 1998). A precise understanding of their morphology is fundamental not only to microbiology but also to environmental science, agriculture, medicine, and food science (Young, 2006). Consequently, establishing a streamlined SEM observation method that preserves microorganisms in their near-native state represents a significant advancement from both academic and applied perspectives (Golding et al., 2016).

By minimizing artifacts, this method enables the high-fidelity evaluation of subtle surface damage and morphological shifts induced by antimicrobial treatments, eliminating the risk of misinterpreting preparation-induced deformations (Carson et al., 2002). Beyond individual cell analysis, this approach preserves the integrity of the extracellular polymeric substance (EPS) matrix (Flemming and Wingender, 2010). Such capability is poised to transform our understanding of complex microbial networks, offering unprecedented insights into quorum-sensing–mediated interactions and the intricate three-dimensional architecture of biofilms (Nadell et al., 2009).

## 5 Conclusion

This study establishes a standardized water freeze-drying (WFD) protocol that ensures high structural fidelity for the SEM observation of bacterial monolayers and sensitive microorganisms. The most significant achievement of this method is the near-total elimination of specimen shrinkage—a long-standing challenge in conventional drying—within a remarkably short processing time of 2–3 h.

Notably, the WFD protocol successfully differentiated the intrinsic mechanical responses of diverse bacterial species. In *E. coli*, WFD revealed previously unrecognized heterogeneity in polar morphologies, including forms flatter than those typically observed by conventional SEM, potentially reflecting native-state morphological heterogeneity. Conversely, in *B. subtilis*, the method preserved the native hemispherical poles, highlighting the structural robustness of the Gram-positive cell wall which remains rigid despite reduced drying stress. These findings demonstrate that WFD provides a more accurate representation of bacterial ultrastructure by preserving the interplay between internal turgor pressure and cell wall rigidity.

Furthermore, we demonstrated that this high-fidelity preservation can be achieved using general-purpose laboratory freeze-dryers, without the need for specialized or high-cost equipment such as high-pressure freezing systems. This accessibility, combined with rapid turnaround and reliability, positions WFD as a practical and cost-effective alternative to existing cryofixation techniques. In conclusion, the WFD protocol provides an accessible alternative for routine ultrastructural analysis, lowering the technical and financial barriers to high-quality SEM imaging and facilitating broader microbial research across diverse scientific disciplines.

## Data Availability

The original contributions presented in the study are included in the article/Supplementary material, further inquiries can be directed to the corresponding author.

## Supplementary material

Supplementary Video S1 Freezing procedure used for water freeze-drying (WFD). Cells suspended in distilled water were frozen according to the procedure described in the Materials and methods. This video illustrates the sample handling during freezing prior to freeze-drying.
